# Comparative analysis of vitamin C derivatives on ATP homeostasis across diverse cell types

**DOI:** 10.1515/biol-2025-1383

**Published:** 2026-09-21

**Authors:** Anna Goc, Waldemar Sumera, Aleksandra Niedzwiecki

**Affiliations:** Dr. Rath Research Institute, 5941 Optical Ct., 95138, San Jose, CA, USA

**Keywords:** vitamin C, ATP, mitochondria, mitobiogenesis

## Abstract

Cellular energy homeostasis is tightly regulated by mitochondrial function and substrate availability. Vitamin C is a redox-active molecule with emerging roles in cellular metabolism; however, the metabolic impacts of structurally distinct vitamin C derivatives remain incompletely defined. In this study, we systematically compared l-ascorbic acid, dehydroascorbic acid, 6-O-palmitoyl-l-ascorbate, and mineral ascorbates (calcium, magnesium, sodium, and potassium salts) across multiple mammalian cell types. Cells were exposed to concentrations ranging from 60 to 500 µM, encompassing physiologically and pharmacologically relevant levels. The vitamin C derivatives induced cell type- and concentration-dependent modulation of intracellular ATP levels. Notably, HepG2 and renal epithelial cells exhibited robust ATP increases, whereas fibroblasts, myocytes, microglia, and skeletal muscle models displayed variable responses depending on the derivative and dose. Moreover, in skeletal muscle cells, co-treatment of standard l-ascorbic acid with select fatty acids did not yield additive bioenergetic effects, suggesting a localized, substrate-specific plateau or shared regulatory step in this specific cell model. Exposure to l-ascorbic acid induced selective increases in COX-1 without changes in SDH-A, consistent with rapid functional modulation of mitochondrial activity in myocytes and microglial cells only. Collectively, these findings demonstrate that vitamin C derivatives modulate cellular ATP levels within existing metabolic networks in a context-dependent manner, consistent with the modulation of mitochondrial-associated energy metabolism.

## 1 Introduction

Mitochondrial ATP production is central to cellular energy homeostasis. In aerobic cells, ATP is generated primarily through oxidative phosphorylation (OXPHOS), in which electrons derived from nutrient oxidation, drive proton pumping across the mitochondrial inner membrane to power ATP synthase [1], [2], [3]. This system integrates substrate availability with mitochondrial respiratory capacity and depends on the structural organization of respiratory complexes within the inner membrane [2], 3]. Disruption of mitochondrial function impairs ATP synthesis and increases reactive oxygen species (ROS) production, contributing to metabolic and degenerative diseases [4], [5], [6]. Despite extensive knowledge of mitochondrial pathways, how redox-active nutrients interact with substrate metabolism to regulate ATP homeostasis remains incompletely understood [5], 7].

Vitamin C (l-ascorbic acid) acts as a redox-active molecule influencing cellular metabolism beyond its classical antioxidant role. It participates in redox cycling and enzyme cofactor functions that can modulate reactive oxygen species (ROS) levels and metabolic signaling pathways, including AMPK-related responses [7], [8], [9], [10]. Experimental studies suggest that vitamin C may support mitochondrial function and ATP production under stress conditions, although these effects are highly context-dependent and may differ in transformed or metabolically altered cells [8], [9], [10], [11], [12], [13], [14], [15]. Compounding these cellular variations, standard l-ascorbic acid is a highly volatile antioxidant; its rapid oxidation and inherent instability in aqueous cell culture media significantly limit its experimental efficacy and mask its true bioenergetic potential [16]. Consequently, exploring structurally distinct analogs – such as the primary oxidized metabolite dehydroascorbic acid, the lipophilic ester 6-O-palmitoyl-l-ascorbate, and buffered mineral salts (calcium, magnesium, potassium, and sodium ascorbate), is necessary to overcome these pharmacokinetic limitations [17], 18]. These variations dictate localized transport mechanics; mineral salts dissociate into active ascorbate and their respective bioenergetic cations, lipid-bound esters directly bypass traditional hydrophilic gating mechanisms to partition into lipid bilayers, and dehydroascorbic acid rapidly hijacks glucose transporters (GLUTs) before being intracellularly recycled back into active ascorbate via glutathione (GSH)- and NADPH-dependent pathways [18], [19], [20], [21].

Cellular ATP output is also strongly influenced by substrate selection. Fatty acid oxidation (FAO) is a high-yield energy pathway regulated by PPARs and PGC-1α and is prominent in oxidatively stressed tissues [22], [23], [24]. However, FAO requires high oxygen consumption and may promote ROS generation under lipid overload [25], 26]. In contrast, glucose metabolism provides more flexible ATP production through glycolysis, particularly under hypoxic conditions [27], [28], [29]. Thus, the ATP homeostasis reflects coordinated regulation of mitochondrial function, substrate availability, and metabolic demand [27], [28], [29].

Glucose metabolism (particularly glycolysis) can turn on and change rate rapidly, making it better suited for sudden or very high-power demands. Fatty acid oxidation is slower to adjust and depends on adequate oxygen, so it tends to dominate at rest, during fasting, and moderate-intensity exercise [30], 31]. Many tissues (e.g., heart, kidney cortex, liver, and certain types of cancers) rely heavily on mitochondrial fatty acid oxidation for baseline ATP, while others (like the brain and red blood cells) depend largely on glucose [32], [33], [34], [35]. These insights underscore the intricate interplay between nutrient metabolism, redox control, and mitochondrial biogenesis. Understanding how modulators such as vitamin C and fatty acid metabolism influence mitochondrial energy production and integrity may provide new avenues for therapeutic intervention in mitochondrial and metabolic disorders. Whether vitamin C-dependent redox signaling intersects with FAO-driven metabolic remodeling to shape mitochondrial ATP production, stress responses, and disease susceptibility remains poorly understood.

Although vitamin C and metabolic substrates independently modulate cellular energy production, it remains unclear whether stable vitamin C formulations intersect directly with substrate-driven mitochondrial pathways or operate via independent, compartmentalized redox mechanisms. We hypothesized that structurally distinct vitamin C derivatives differentially modulate ATP production in a cell type-dependent manner by altering mitochondrial activity and interacting with substrate availability in a context-specific way. To test this, we systematically assessed ATP levels and mitochondrial-associated markers across several human and rodent cell models, representing distinct malignant and non-malignant metabolic phenotypes exposed to this diverse profile of vitamin C derivatives, under defined substrate conditions. These findings clarify the dual role of vitamin C as both a direct free-radical scavenger and an essential reducing co-factor for iron-containing Fe(II)/2-oxoglutarate-dependent dioxygenases, establishing a clearer link between nutrient metabolism, transport kinetics, and mitochondrial integrity [16], 36].

## 2 Materials and methods

### 2.1 Materials

Human normal dermal fibroblasts (HNDF), human hepatocellular carcinoma cells (HepG2), the immortalized rat skeletal myoblast cell line (L6), the human immortalized ventricular cardiomyocyte-like cell line (AC16), the human epithelial kidney cell line (HEK), and undifferentiated human skeletal muscle cells (SkM) were obtained from the ATCC (Manassas, VA, USA). Except for SkM, all cells were maintained in Dulbecco’s Modified Eagle’s Medium (DMEM) from ThermoFisher Scientific (Waltham, MA, USA) supplemented with 10 % Fetal Bovine Serum (FBS) from ThermoFisher Scientific (Waltham, MA, USA) and 1 % Penicillin-Streptomycin (PS) from MilliporSigma (St. Louis, MO, USA). Human skeletal muscle cells (non-differentiated) were isolated from normal, human skeletal muscle, and maintained in Mesenchymal Stem Cell Basal Medium supplemented with the Primary Skeletal Muscle Growth Kit from the ATCC (Manassas, VA, USA). For differentiation, undifferentiated SkM were cultured in single-component Skeletal Muscle Differentiation Tool from the ATCC (Manassas, VA, USA) for 15 days. Mouse microglial cells (IMG) were obtained from Kerafast (Newark, CA, USA). It is an immortalized microglial cell line isolated from the brains of adult mice. IMG cells were maintained in DMEM supplemented with 10 % FBS and 1 % PS from MilliporeSigma (St. Louis, MO, USA). All forms of ascorbic acid except for dehydroascorbic acid that was obtained from Cayman Chemical (Ann Arbor, MI, USA), were purchased from MilliporSigma (St. Louis, MO, USA) and dissolved at 6.0 mM concentration in 1 × PBS, with the exception of 6-O-palmitoyl-l-ascorbate (A6P) that was dissolved in 10 % dimethyl sulfoxide (DMSO) purchased from MilliporSigma (St. Louis, MO, USA). The final DMSO concentration in culture media did not exceed 0.2 % in any experiment. All solutions were sterilized with a 0.2 µm filter. Other compounds such as fatty acids, glucose, fructose, and taurine were purchased from Cayman Chemical (Ann Arbor, MI, USA).

### 2.2 Methods

#### 2.2.1 Cell culture and treatment workflow

To eliminate confounding serum-derived growth factors and mitigate metal-ion-induced auto-oxidation of the ascorbic acid formulations in the extracellular matrix, the vitamin- and mineral-free Minimum Essential Medium Eagle (MEM) was used. Cells were plated in MEM supplemented with 10 % FBS, then washed twice with sterile 1 × phosphate-buffered saline (1 × PBS) and replenished with completely serum-free MEM (i.e., 0 % FBS) containing the designated concentrations of vitamin C derivatives or benchmarking substrates for a standardized acute treatment window only.

#### 2.2.2 ATP production assay

Cells were plated on 96-well plates in vitamin- and mineral-free MEM supplemented with 10 % FBS at 1.0 × 10^5^ density for 8 h to allow cell attachment. Next, the culture medium was replaced with serum-free MEM and different forms of vitamin C were applied at final concentrations of 60 µm, 90 µM, 120 µM, 250 µM, and 500 µM for 12 h at 37 °C. Following incubation, the conditioned medium from all wells was discarded and cells were subjected to ATP bioluminescence assays to quantify the ATP amount using an ATP Bioluminescence Assay Kit (MilliporSigma, St. Louis, MO, USA) according to the provided protocol. The ATP detection mix was diluted at 1:4, added to all wells, and luminescence measurements were performed immediately to assess relative chemiluminescence units (RLU) using a multimode plate reader (Tecan Group Ltd., Switzerland). Results are expressed as a percentage of the experimental agent-free control (mean ± SD, data were normalized to untreated controls per cell number).

#### 2.2.3 Mitochondrial biogenesis assay

MitoBiogenesis™ In-Cell ELISA Kit (Colorimetric) was purchased from Abcam (Cambridge, UK) and used to examine mitochondrial biogenesis in select cell types. Briefly, cells were grown to confluency in 96-well plates in vitamin- and mineral-free MEM supplemented with 10 % FBS, then replaced with serum-free MEM and treated with test ingredients for 12 h at 37 °C. Media were removed and cells were washed with 1 × PBS provided in the kit. Next, cells were fixed and processed according to the protocol provided by the manufacturer. This assay was used to quantify two mitochondrial proteins: subunit I of Complex IV (COX-1) and the 70 kDa subunit of Complex II (SDH-A). Results are expressed as a percentage of the experimental agent-free control (mean ± SD, data were normalized to untreated controls per cell number).

#### 2.2.4 Viability assay

A 3-[4,5-dimethylthiazol-2-yl]-2,5 diphenyl tetrazolium bromide (MTT) assay was used to assess cell viability. Briefly, cells were seeded into 96-well plates in vitamin- and mineral-free MEM supplemented with 10 % FBS at a cell density of 4 × 10^4^ per well and allowed to adhere overnight. Then, culture medium was replaced with serum-free MEM and cells were treated respectively with designated concentrations of the tested compounds for 12 h. Next, the complete growth medium was replaced with a fresh medium containing 5.0 mg/ml MTT, followed by incubation for 3 h at 37 °C. After removing the culture medium, 100 μl of methanol was added and the absorbance was measured at 570 nm using a microplate spectrophotometer (Molecular Devices, San Jose, CA). Results are expressed as a percentage of the experimental agent-free control.

### 2.3 Statistical analysis

All quantitative data are expressed as mean ± standard deviation (SD) compiled from a minimum of three independent biological replicates (*n* = 3), with each individual experiment performed in technical quadruplicate and averaged prior to statistical analysis. Statistical evaluations were executed using GraphPad Prism software (GraphPad Software, Boston, MA, USA). For comparisons involving a single independent variable across multiple experimental groups, a one-way analysis of variance (ANOVA) followed by Dunnett’s *post hoc* test or Tukey’s *post hoc* multiple comparisons test was utilized. For complex datasets involving multiple independent variables specifically, a two-way ANOVA followed by Dunnett’s *post hoc* test was applied to compare each treatment concentration and derivative directly back to its respective untreated control group. Pairwise comparisons between a single treated group and a control (where applicable) were evaluated using a two-tailed Student’s *t*-test. A *p*-value of <0.05 was defined as the threshold for statistical significance across all analyses.

## 3 Results

### 3.1 Effects of vitamin C derivatives on ATP production in human and rodent cells

Treatment of selected human and rodent cell lines with multiple vitamin C derivatives, including l-ascorbic acid, 6-O-palmitoyl-l-ascorbate, and mineral ascorbates (calcium, magnesium, sodium, and potassium), as well as dehydroascorbic acid, significantly modulated intracellular ATP levels across concentrations ranging from 60 to 500 µM. The effect of these derivatives at their highest concentrations on viability of differentiated SkM cells are described below in Section 3.2.

**Figure 1:**
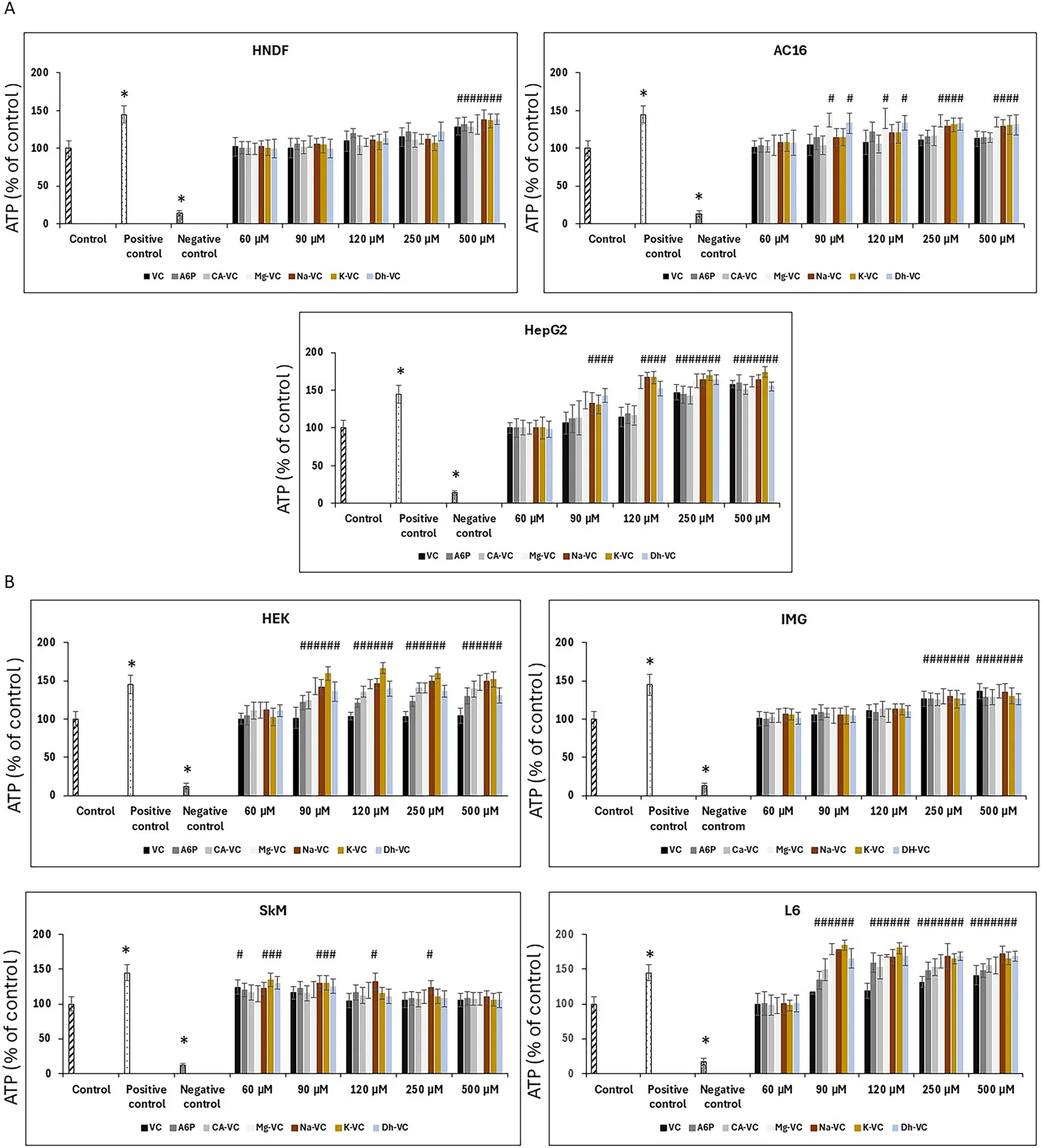
Effect of different forms of vitamin C on ATP production *in vitro*. (A) Effect of different forms of vitamin C on ATP production in human normal dermal fibroblasts (HNDF), human myocytes (AC16), and human hepatoma cell line (HepG2). Cells were treated with various forms of vitamin C at concentrations ranging from 60 to 500 µM for 12 h and subjected to ATP production ELISA assay. (B) Effect of different forms of vitamin C on an ATP production in human epithelial kidney cells (HEK), immortalized mouse glial cells (IMG), differentiated human skeletal muscle cells (SkM), and rat myoblasts derived from skeletal muscle (L6). Cells were treated with various forms of vitamin C at concentrations ranging from 60 to 500 µM for 12 h and subjected to ATP production ELISA assay. Control – 0.02 % DMSO, positive control – ATP solution provided by manufacturer, negative control – 100 % dead cells. VC – vitamin C (l-ascorbic acid), A6P – 6-O-palmitoyl-l-ascorbate, Ca-VC – calcium ascorbate, Mg-VC – magnesium ascorbate, Na-VC – sodium ascorbate, K-VC – potassium ascorbate, Dh – dehydroascorbic acid. Data are expressed as mean ± SD compiled from *n* = 3 independent biological experiments, with technical quadruplicates averaged prior to analysis. Statistical significance was determined via a two-way ANOVA followed by Dunnett’s *post hoc* test to correct for multiplicity. Significance symbols indicate a statistical deviation from the untreated control group #*p* < 0.05, &*p* < 0.01, *p < 0.001.

In HNDF all tested derivatives applied only at 500 µM caused a significant increase in ATP levels (28–38 %). In AC16 cells, magnesium ascorbate induced the strongest response, elevating ATP production by 34–40 %, while dehydroascorbic acid increased ATP by 21–31 % across 90–500 µM concentrations. Sodium and potassium ascorbates, respectively, triggered moderate 20–31 % increases at 250–500 µM concentrations. In HepG2 cells, several derivatives, including magnesium, sodium, and potassium ascorbates as well as dehydroascorbic acid, enhanced ATP production by 36–43 % at 90 µM, with further 51–73 % increases observed at 120–500 µM concentrations. l-ascorbic acid, palmitoyl-ascorbate, and calcium ascorbate also elevated ATP levels by 43–49 % at 250–500 µM concentrations (Figure 1A).

In HEK cells, all derivatives except l-ascorbic acid significantly increased ATP levels. Palmitoyl-ascorbate and calcium ascorbate increased ATP by 21–39 % and 23–40 %, respectively, whereas magnesium, sodium, potassium ascorbates as well as dehydroascorbic acid produced larger 30–66 % increases across 90,500 µM concentrations. IMG cells exhibited a consistent increase in ATP levels (i.e., 25–30 %) following treatment with all vitamin C forms at 250–500 µM concentrations. In SkM cells, l-ascorbic acid increased ATP by 24 % at 60 µM only, whereas sodium and potassium ascorbates and dehydroascorbic acid elevated ATP by 22–34 % at 60–90 µM concentrations and by approximately 23–31 % at 120–250 µM concentrations. In L6 cells, l-ascorbic acid increased ATP levels by 31–41 % at 250–500 µM concentrations. Palmitoyl-ascorbate and calcium ascorbate caused ATP increases of 34–54 % at 90–500 µM concentrations, while magnesium, sodium, potassium ascorbates and dehydroascorbic acid elicited the strongest 55–78 % effects over the same concentration range (Figure 1B).

### 3.2 Comparison with other metabolic substrates in human skeletal muscle cells

**Figure 2:**
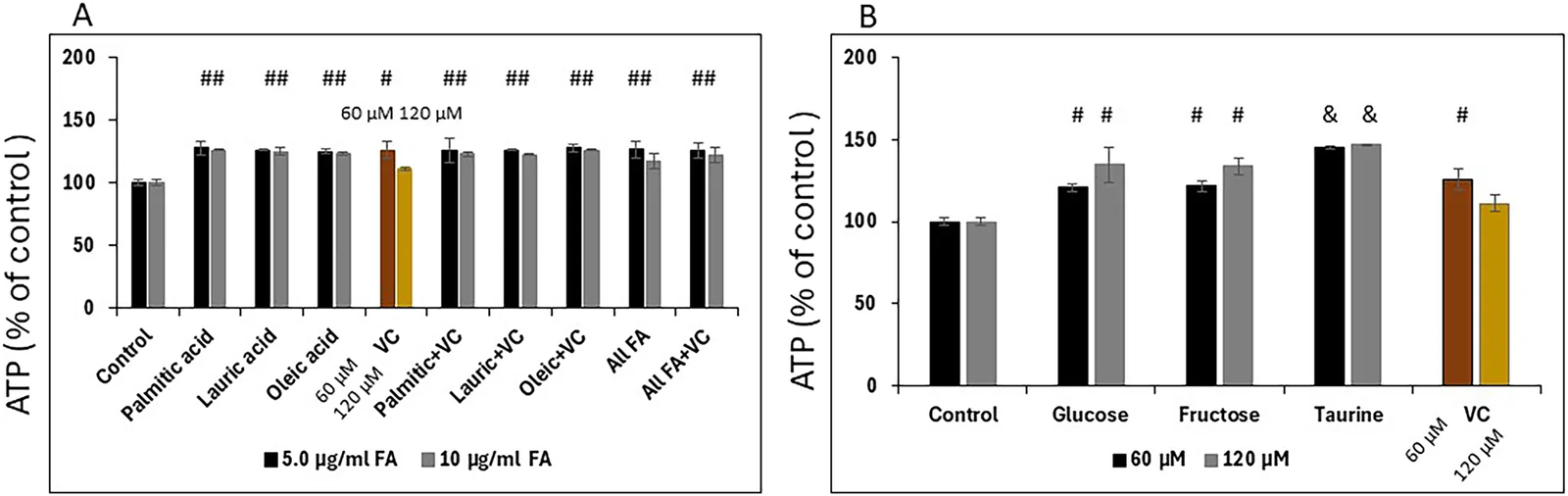
Effects of fatty acids on ATP production *in vitro*. (A) Effect of palmitic acid, lauric acid, and oleic acid alone or their combination with vitamin C on ATP production in human differentiated skeletal muscle cells (SkM). Cells were treated with select fatty acids individually at concentrations of 5.0 and 10 µM or in combinations with 60 µM and 120 µM vitamin C for 12 h and subjected to ATP production ELISA assay. (B) Effect of different compounds on an ATP production in human differentiated skeletal muscle cells (SkM). Cells were treated with glucose, fructose, and taurine, respectively, at 60 µM and 120 µM concentrations for 12 h and subjected to ATP production ELISA assay. Control – 0.02 % DMSO. Data are expressed as mean ± SD compiled from *n* = 3 independent biological experiments, with technical quadruplicates averaged prior to analysis. Statistical significance was determined via a two-way ANOVA followed by Dunnett’s *post hoc* test to correct for multiplicity. Significance symbols indicate a statistical deviation from the untreated control group #*p* < 0.05, &*p* < 0.01.

**Figure 3:**
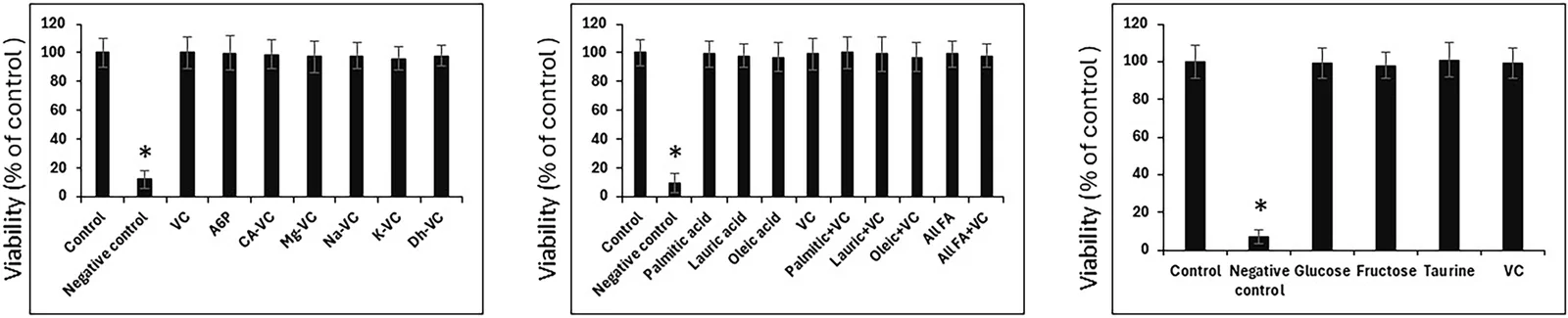
Evaluation of different test compounds on viability of human differentiated skeletal muscle cells. Cells were treated with 500 µM vitamin C, select fatty acids (at 10 μg/ml) individually and in combinations with 500 µM vitamin C, as well as glucose, fructose, and taurine at 60 µM for 12 h, followed by the assessment of their viability using the MTT method and measuring absorbance at 570 nm. Control – 0.02 % DMSO, negative control – 100 % dead cells. VC – vitamin C (l-ascorbic acid), A6P – 6-O-palmitoyl-l-ascorbate, Ca-VC – calcium ascorbate, Mg-VC – magnesium ascorbate, Na-VC – sodium ascorbate, K-VC – potassium ascorbate, Dh – dehydroascorbic acid, FA – fatty acid. Data are expressed as mean ± SD compiled from *n* = 3 independent biological experiments, with technical quadruplicates averaged prior to analysis. Statistical significance was determined via a one-way ANOVA followed by Dunnett’s *post hoc* test to correct for multiplicity. Significance symbols indicate a statistical deviation from the untreated control group *p < 0.001.

To further evaluate the metabolic effects of l-ascorbic acid, ATP production was compared with that induced by alternative energy substrates in human differentiated skeletal muscle cells. Fatty acids (palmitic, lauric, and oleic acids) increased ATP levels by 21–26 % at 5.0 μg/ml (i.e., 19.5 µM palmitic acid, 25.0 µM lauric acid, 17.7 µM oleic acid) and 25–27 % at 10 μg/ml (i.e., 39.0 µM palmitic acid, 50.0 µM lauric acid, 35.4 µM oleic acid) (Figure 2A). Co-treatment with these respective fatty acids and 60 μM l-ascorbic acid produced only marginal additional effects. l-ascorbic acid alone significantly increased ATP production at 60 µM to levels comparable to those induced by fatty acids. However, a slight reduction was observed at 120 µM. Glucose and fructose induced modest but statistically significant concentration-dependent increases in ATP levels (i.e., 20–34 %), whereas taurine produced a more pronounced effect, increasing ATP levels by 45–46 % at both 60 µM and 120 µM concentrations (Figure 2B). Notably, within the parameters of our skeletal muscle model, the combination of l-ascorbic acid and select fatty acids failed to produce an additive “boost” in intracellular ATP, indicating that under these localized conditions, these pathways may intersect or saturate. No cytotoxic effects were observed in differentiated SkM cells following treatment with any of the tested compounds at indicated concentrations (Figure 3).

### 3.3 Effect of vitamin C on acute modulation of COX-1 and SDH-A signal intensity

**Figure 4:**
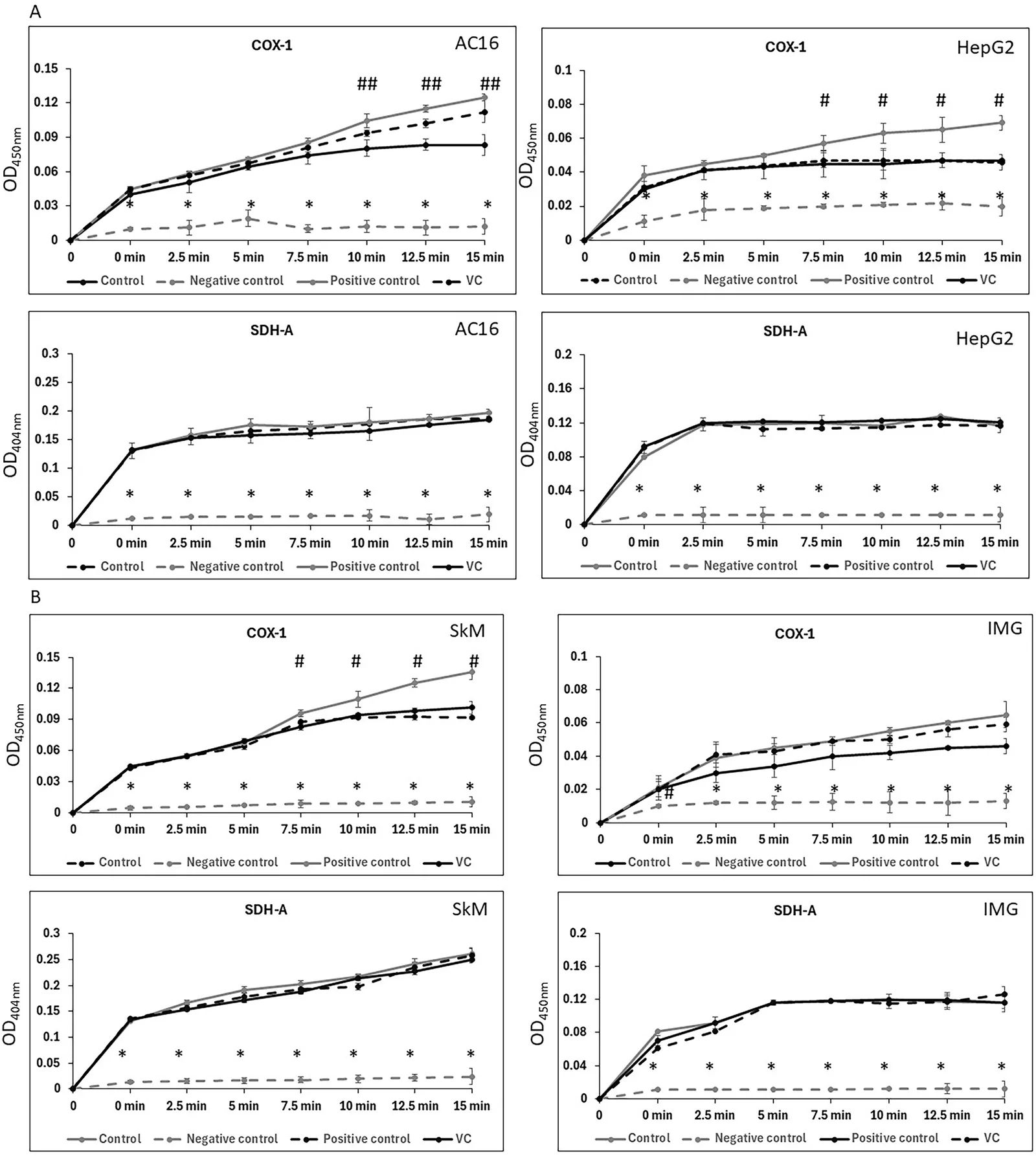
Effect of vitamin C on mitochondrial biogenesis *in vitro*. (A) Effect of vitamin C on mitochondrial biogenesis in human myocytes (AC16) and the human hepatoma HepG2 cell line. Cells were grown to confluency and treated with vitamin C at 60 µM for 12 h. The cells were then subjected to kinetic mitobiogenesis ELISA assay as described in Materials and Methods. This assay was used to quantify mitochondrial DNA coded protein subunit 1 of complex IV (COX-1) and nuclear DNA coded protein SDH-A. (B) Effect of vitamin C on mitochondrial biogenesis in human differentiated skeletal muscle cells (SkM) and immortalized mouse glial cells (IMG). Cells were grown to confluency and treated with vitamin C at 60 µM for 12 h. The cells were then subjected to kinetic mitobiogenesis ELISA assay as described in Materials and Methods. Control – 0.02 % DMSO, negative control – 100 % dead cells, positive control – 60 µM taurine as metabolic positive control. Data are expressed as mean ± SD compiled from *n* = 3 independent biological experiments, with technical quadruplicates averaged prior to analysis. Statistical significance was determined via a two-way ANOVA followed by Dunnett’s *post hoc* test to correct for multiplicity. Significance symbols indicate a statistical deviation from the untreated control group #*p* < 0.05, &*p* < 0.01, *p < 0.001.

To evaluate the immediate impacts of the vitamin C formulations on key respiratory chain proteins, we monitored mitochondrial DNA-encoded cytochrome *c* oxidase subunit I (COX-1) and nuclear DNA-encoded succinate dehydrogenase subunit A (SDH-A) levels following an acute 15-min treatment window using a kinetic ELISA capture system. Treatment with 60 μM l-ascorbic acid selectively increased COX-1 protein levels in AC16 cells and IMG cells by 43 % and 24 %, respectively, within 15 min, without affecting SDH-A expression (Figure 4A). No significant changes in COX-1 or SDH-A levels were observed in other cell types examined, including SkM cells, L6 cells, and HepG2 cells (Figure 4B). Crucially, this observed, rapid 43 % increase in detectable COX-1 signal intensity within this timeframe cannot be attributed to true *de novo* mitochondrial biogenesis or active protein translation, as the 15-min window is far too short to accommodate the transcription and translation of mitochondrially encoded genes. Instead, this acute increase strictly reflects physical or assay-driven phenomena, such as treatment-induced alterations in protein stability, rapid modifications to the labile mitochondrial protein pool, or conformational shifts that enhanced antibody epitope exposure upon cell lysis. Therefore, these specific data should be interpreted strictly as an indicator of acute, structural or biophysical protein stabilization rather than an expansion of the total mitochondrial network.

## 4 Discussion

In this study, we tested whether structurally distinct vitamin C derivatives modulate cellular ATP production in a cell type-dependent manner and whether these effects interact with substrate-driven metabolic pathways. Across multiple human and rodent cell systems, vitamin C derivatives consistently altered ATP levels within a physiologically and pharmacologically relevant concentration range [11], 37]. The magnitude and dose dependence of these responses varied across both cell types and compound structures, suggesting cell-type–dependent modulation of ATP level**s** in a context-dependent manner rather than producing uniform metabolic effects.

ATP responses differed markedly across cell types. HepG2 cells and HEK cells exhibited robust ATP increases across the tested vitamin C derivatives, consistent with differences in their mitochondrial and metabolic characteristics [3], 6]. HNDF also showed strong responses but only at highest 500 µM concentration, whereas IMG cells displayed more modest increases, suggesting perhaps rather constrained ATP responses in immune-lineage cells under these conditions [7]. Skeletal muscle models revealed both species- and state-dependent differences. Rat L6 cells exhibited the strongest responses overall, whereas differentiated human SkM cells showed more moderate and non-linear behavior, including reduced ATP responses at intermediate and the highest l-ascorbic acid concentrations. This non-linear behavior may reflect complex dose-dependent dynamics rather than simple concentration-driven stimulation, potentially involving transport mechanisms, redox states, or metabolic pathway contributions [7], 10]. Comparison with classical metabolic substrates revealed that fatty acids, glucose, fructose, and taurine all increased ATP levels within a similar or overlapping range as vitamin C derivatives [27], [28], [29]. Taurine produced the largest and consistent increase under the conditions tested. These finding position taurine as a comparatively strong ATP-increasing compound under these experimental settings and suggests that vitamin C derivatives act within a limited range of ATP responses similar to other metabolic substrates.

While our observations in SkM cells hint at a non-additive relationship when pairing standard l-ascorbic acid with select fatty acids, these results must be interpreted with caution. Because this interaction was mapped using a single derivative and cell line, further research across a broader panel of malignant cell types and lipophilic formulations (such as 6-O-palmitoyl l-ascorbate) is required to determine if this metabolic intersection is generalizable or unique to differentiated muscle bioenergetics. This lack could also further supports the idea that these interventions act within overlapping metabolic processes rather than through additive mechanisms [27], [28], [29]. Collectively, these results may also indicate that cellular ATP output is constrained by substrate utilization, mitochondrial capacity, and/or energy demand, rather than additive contributions from individual metabolic inputs [2], [27], [28], [29]. However, direct measurements of metabolic flux would be required to determine whether these compounds act on shared or distinct pathways and substantiate these suggestions. It is worth mentioning that in non-stressed systems, vitamin C is generally understood to preserve mitochondrial integrity and support enzymatic activity, while more pronounced metabolic effects are often observed under conditions of oxidative stress or metabolic dysfunction. High pharmacological concentrations, particularly in cancer models, have been associated with altered metabolic flux and ATP depletion, highlighting the context-dependent nature of bioenergetic effects of vitamin C [38], [39], [40].

Exposure to l-ascorbic acid was associated with increased COX-1 protein levels in selected cell types without corresponding changes in SDH-A. Given the 12 h exposure time of this compound on cells and rapid stimulatory response of COX-1 protein, this finding might be consistent with canonical mitochondrial biogenesis but also may reflect swift changes in protein stability, turnover, or post-translational regulation of mitochondrial components [8], 9]. Notably, increases in COX-1 were not consistently accompanied by increases in ATP, and ATP changes were also observed in the absence of detectable alterations in mitochondrial markers. These observations suggest that changes in individual mitochondrial proteins do not necessarily correspond to proportional changes in overall respiratory function. Consistent with established approaches to assessing mitochondrial function, isolated protein measurements provide limited insight into respiratory capacity or coupling efficiency in the absence of functional assays [40], 41]. These findings support a model in which vitamin C derivatives influence mitochondrial function, suggest rapid cellular responses affecting mitochondrial-associated proteins that precede or occur independently of changes in mitochondrial content. A pattern when ATP level is unchanged, but COX-1 is elevated could be explained that agent is acting as a signal to remodel the mitochondrial network (i.e., biogenesis), but the cells are either in an adaptive/transitional state or redistributing energy production pathways and demands so that total ATP does not increase. A pattern when ATP level is elevated but COX-1 is unhanged could be described when an agent is primarily acting as a functional modulator improving mitochondrial efficiency and/or cellular energy balance without triggering a full biogenesis response under applied conditions.

Vitamin C derivatives exhibited distinct activity profiles across cell types. Mineral ascorbates (magnesium, sodium, potassium, calcium) consistently produced strong ATP responses in metabolically active cells, suggesting that associated cations may contribute to differences in cellular responses under these conditions, consistent with established roles of mineral cofactors in bioenergetics regulation [42]. For example, potassium is essential for maintaining mitochondrial membrane potential, while calcium and magnesium provide important cofactors for the activity of several tricarboxylic acid cycle (TCA) enzymes, including rate-limiting steps and respiratory enzymes, as well as affecting bioenergetics and redox state [42]. Lipophilic palmitoyl-ascorbate showed concentration- and cell-type selective effects, suggesting that its structural property enhancing membrane affinity may influence cellular uptake or distribution and downstream metabolic effects, potentially explaining its differential potency compared with hydrophilic derivatives [10], 12]. Dehydroascorbic acid also produced strong ATP increases across several models, consistent with its distinct transport and intracellular reduction dynamics, which may be associated with but not limited to redox-dependent intracellular effects or processes that could influence metabolism [12].

Taken together, these findings support a model in which vitamin C derivatives modulate ATP production through context-dependent interactions with mitochondrial function, redox state, and substrate availability. Rather than acting as direct ATP-generating substrates, their effects appear to produce similar directional effects on ATP levels as classical metabolic substrates do, including glucose, fatty acids, and taurine [27], [28], [29]. The lack of additive effects with classical substrates and the variability across models support the interpretation that ATP levels reflect the integration of multiple metabolic processes rather than simple summation of individual inputs [27], [28], [29]. The concept of an “energetic ceiling” may provide a useful framework for interpreting these observations, although this was not directly tested and requires further investigation. The observed non-linearity in dose responses, strong cell-type dependence, and lack of additive effects with classical substrates all indicate that ATP production operates within a regulated context, where modulation occurs through redistribution and optimization of existing metabolic capacity rather than expansion of maximal output.

Intracellular measurements of ATP levels provide a useful but less specific readout of cellular energy status as they do not distinguish between mitochondrial and glycolytic contributions [41]. In addition, our mitochondrial analysis was limited to COX-1 and SDH-A, and no direct measurements of respiratory flux, coupling efficiency, or substrate oxidation rates were performed [43]. Future studies incorporating real-time flux analysis, isotope tracing, and/or oxygen consumption measurements will be necessary to define pathway-specific contributions. The rapid COX-1 response also warrants further investigation into non-transcriptional mitochondrial regulation mechanisms [8], 9]. A key finding requiring a rigorous technical caveat is the rapid, 15-min escalation of detectable COX-1 protein levels following derivative treatment. While the Abcam MitoBiogenesis™ In-Cell ELISA platform utilizes a COX-1/SDHA ratio for monitoring long-term ‘mitochondrial biogenesis,’ we must plainly state that true biogenesis was not, and could not be, measured within this acute timeframe. According to the manufacturer’s parameters, detecting genuine biogenesis requires tracking treatments across multiple cell doublings. The translation of core mtDNA-encoded respiratory chain subunits like COX-1 is highly rate-limited on mitochondrial ribosomes. Thus, rather than indicating transcriptional or translational induction, our data indicate that vitamin C derivatives prompt immediate non-transcriptional changes to existing mitochondrial protein pools. These alterations likely involve the rapid stabilization of specific complexes against degradation, or conformational changes associated with respiratory super-complex assembly or disassembly that unmask antibody-binding epitopes during the Triton X-100 permeabilization and fixation steps. Future studies utilizing cytoplasmic translation inhibitors like cycloheximide or mitochondrial translation inhibitors like chloramphenicol are mandatory to definitively separate these biophysical assay kinetics from true structural mitochondrial modifications.

The distinct metabolic responses observed among the evaluated cell lines are dictated by the specific transport kinetics of each vitamin C formulation. Reduced mineral salts (sodium, potassium, calcium, and magnesium ascorbate) rely primarily on sodium-dependent active transport via SVCT1 and SVCT2, whereas the oxidized metabolite dehydroascorbic acid is internalized rapidly via facilitated diffusion through GLUTs. In glycolytic cancer cell lines exhibiting elevated baseline GLUT expression, the sudden influx of dehydroascorbic acid causes a metabolic shift; immediate recycling of dehydroascorbic acid back to ascorbate consumes intracellular GSH and NADPH [34], 44]. This transient redox drain restricts downstream glycolysis by inactivating glyceraldehyde-3-phosphate dehydrogenase (GAPDH) via oxidative modification [45]. Conversely, the non-cancerous control fibroblasts maintain balanced transport machinery and physiological redox buffers, preventing this sudden metabolic collapse [20], 45]. Furthermore, the lipophilic ester 6-O-palmitoyl-l-ascorbate bypasses traditional hydrophilic gating mechanisms altogether. This structural modification allows it to interact directly with cellular lipid bilayers, facilitating direct intracellular delivery independent of standard transport pathways [18], 19]. Beyond transport variations, internalized ascorbate serves as an essential reducing co-factor for the iron-containing Fe(II)/2-oxoglutarate-dependent dioxygenase superfamily. Consequently, cancer cell lines, which feature accelerated metabolic rates, exhibit heightened sensitivity to these treatments compared to fibroblasts, a vulnerability mediated by the accelerated hydroxylation of HIF-1α and subsequent downregulation of downstream glycolytic gene targets [36], 45]. Concurrently, variations in intracellular iron status, specifically the magnitude of the baseline labile iron pool (LIP), dictate whether the formulation acts as a protective antioxidant or a metabolic pro-oxidant. In non-cancerous cells, homeostatic ascorbate levels support standard tricarboxylic acid cycle efficiency [20]. However, in cancer cells with elevated baseline oxidative stress or expanded LIPs, high intracellular ascorbate concentrations induce localized hydrogen peroxide generation via Fenton chemistry [46]. This selectively compromises mitochondrial membrane integrity, suppressing oxygen consumption rates (OCR) and inducing a metabolic energy crisis specifically in the tumor model while sparing control cells [36].

## 5 Conclusions

In summary, our results support the observation that vitamin C derivatives affect ATP levels in a cell type- and structure-dependent manner through context-dependent metabolic effects involving mitochondrial and substrate-related processes. These effects might converge with classical metabolic substrates, including fatty acids, glucose, and taurine, within a shared energetic pathways and processes. However, this warrants further study. Rather than acting as uniform metabolic enhancers, vitamin C derivatives seem to function as factors that influence cellular ATP levels, with effects determined by cellular conditions and compound structure. From a translational perspective, these findings suggest that optimization of vitamin C formulations may influence cellular bioenergetics, particularly in contexts where metabolic balance is altered. However, extrapolation beyond *in vitro* systems should be approached with caution and further work is required to establish physiological relevance in intact tissues and organs.
